# CACHE Challenge #1: Docking with GNINA Is All You Need

**DOI:** 10.1021/acs.jcim.4c01429

**Published:** 2024-12-10

**Authors:** Ian Dunn, Somayeh Pirhadi, Yao Wang, Smmrithi Ravindran, Carter Concepcion, David Ryan Koes

**Affiliations:** Department of Computational and Systems Biology, University of Pittsburgh, 4200 Fifth Ave, Pittsburgh, Pennsylvania 15260, United States

## Abstract

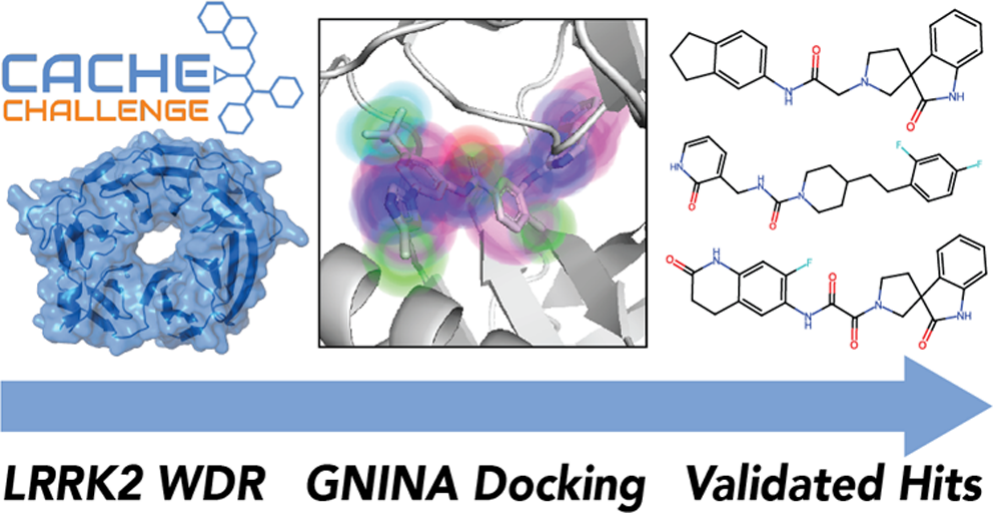

We describe our winning submission to the first Critical Assessment of Computational Hit-Finding Experiments (CACHE) challenge. In this challenge, 23 participants employed a diverse array of structure-based methods to identify hits to a target with no known ligands. We utilized two methods, pharmacophore search and molecular docking, to identify our initial hit list and compounds for the hit expansion phase. Unlike many other participants, we limited ourselves to using docking scores in identifying and ranking hits. Our resulting best hit series tied for first place when evaluated by a panel of expert judges. Here, we report our top-performing open-source workflow and results.

## Introduction

The Critical Assessment of Computational Hit-Finding Experiments (CACHE) is a series of prospective benchmarking challenges in which participants use computational methods to predict up to 100 candidate ligands for a specified target that are then experimentally tested for binding.

**Figure 1:**
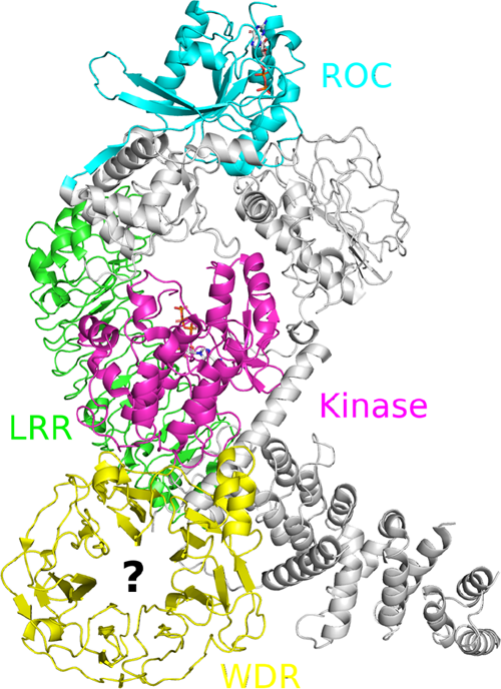
LRRK2 structure (PDB: 7LI4(8)) with domains labeled. The target of the first CACHE challenge was the central cavity of the WDR domain (yellow).

The target for the first CACHE challenge^1^ was the WD-40 repeat (WDR) domain of the Leucine-rich repeat serine/threonine-protein kinase 2 (LRRK2) protein. LRRK2 is a large, multifunctional, multidomain protein that is implicated in the occurrence of familial Parkinson’s Disease (PD).^2^ The predominant hypothesis regarding the role of LRRK2 in PD is that mutations lead to hyperactivity of the kinase domain.^2,3^ As a result, most drug discovery efforts have focused on developing LRRK2 kinase inhibitors.^2,3^ Other strategies have also been pursued, such as degradation via PROTAC compounds.^4,5^ Targeting the WDR domain of LRRK2 is an alternate, yet previously unexplored, avenue for the development of therapeutics for PD. WDR domains are known generally to be disease-associated and druggable.^6^ Recent work suggests the WDR domain plays an important role in the biological function of LRRK2, either through recruiting binding partners or facilitating association with microtubules.^7^ Prior to the CACHE challenge, there were no known ligands for this domain. CACHE participants were tasked with using the apo structure of the WDR domain to identify ligands that bind to its central cavity (see Figure 1).

We present a detailed description of our participation in the first CACHE challenge. Our submission resulted in the identification of two novel chemical series with experimentally confirmed binding to the target and was identified as one of the most promising submissions out of 23 total participants by a panel of independent judges with experience in biophysics, medicinal chemistry, and computational chemistry.

## Methods

**Figure 2:**
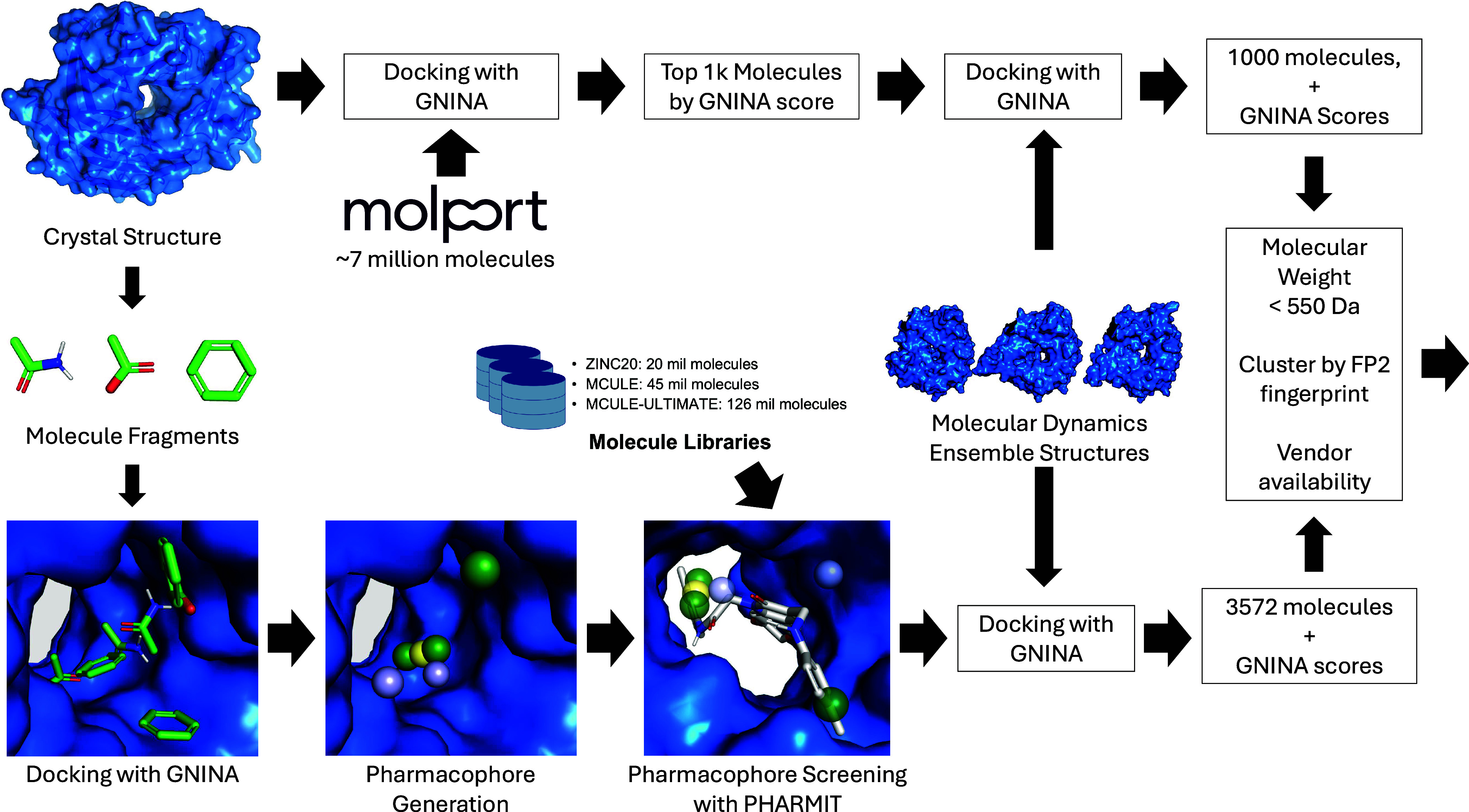
Hit identification workflow. Two approaches, high-throughput molecular docking and pharmacophore screening, were used to identify an initial hit list for submission to the CACHE competition.

Our approach, shown in Figure 2, was composed of standard, structure-based drug discovery methods and used only open-source software. The most prominent of the tools used is Gnina, an open-source molecular docking software that uses a deep-learning-based scoring function to prioritize ligands.^9,10^ For the initial hit-finding round, our hit discovery pipeline was composed of two distinct arms: large-scale docking and pharmacophore screening. In the first arm, we docked 7 million molecules using Gnina. In the latter arm, we used pharmacophore screening to identify the candidate ligands. This two-arm approach resulted in two hits, as classified by the CACHE organizers. In the hit expansion round, we evaluated analogues for one of these hits. Analogues were identified by a similarity search. In both the hit discovery and hit expansion rounds, molecules were selected for testing via ranking with docking scores, with little filtering and no manual curation used to select the final molecules.

### Target Structure

The target for our structure-based approach was an apo (unbound) crystal structure of the WDR domain of the LRRK2 protein (PDB ID: 6DLO).^7^ This 2.7 Å resolution X-ray crystal structure was supplemented with snapshots taken from AMBER20^11^ molecular dynamics simulations of the crystal structure. The system was explicitly solvated and neutralized with sodium ions using tleap.^12^ For the initial equilibration, the system was subject to two rounds of energy minimization: the first with restraints on the protein, 1 ns of NTV molecular dynamics where the protein was restrained and the system was heated from 0 to 300 K, and then a further 1 ns of unrestrained isothermal–isobaric (NTP) simulation at 300 K and 1 atm. The AMBER ff15ipq force field^13^ with TIP3P water was used due to its ability to reproduce nuclear magnetic resonance (NMR) experimental observables.^14^ Following system preparation, ten 100 ns production simulations were performed. A representative ensemble of 5 structures was extracted from these simulations by a greedy clustering algorithm based on pairwise RMSD of MD frames.^15^ Our final ensemble of apo structures consists of the crystal structure and 5 representative poses from MD. These MD-based poses are later referred to as apo-MD-[1–5] in the Results section.

### Round 1: Hit Discovery

For the first round of the challenge, participants were expected to identify a list of 100 commercially available compounds for acquisition and testing that were predicted to bind to the WDR domain. Participants were initially suggested to screen the ENAMINE REAL library. However, due to the Russian invasion of Ukraine, we opted to screen commercial libraries from non-Ukrainian providers due to uncertainty at the beginning of the challenge of the availability of ENAMINE compounds. Our strategy was a two-pronged approach: large-scale docking and pharmacophore screening.

#### Large-Scale Docking

We docked the entire Molport database (https://www.molport.com/) into the 6DLO crystal structure. Compounds were sourced from an early 2022 snapshot of the Molport aggregated library of commercially available compounds containing 7 million compounds (the same set used in Ragoza et al.^16^) Initial conformations were generated using the EmbedMultipleConfs method from RDKit followed by UFF energy minimization. The only protein structure preparation was the removal of water. Protonation was determined within Gnina using OpenBabel.^17^

All molecular docking was done with Gnina,^9^ an open-source molecular docking software. Gnina is a fork of SMINA^18^ which is a fork of AutoDock Vina.^19^Gnina uses convolutional neural networks (CNNs) applied to a grid-based representation^20^ of atomic positions to score ligand poses. By default, Gnina uses the empirical AutoDock Vina scoring function to run Markov Chain Monte Carlo (MCMC) sampling and then uses the CNN scoring function to rescore poses obtained from MCMC. As a result, a docked ligand pose from Gnina is returned with both scores from AutoDock Vina and the Gnina CNN scoring function. The CNN scoring function returns two scores: a binary classification of good/bad pose (probability of being ≤2 Å RMSD from the experimental structure) called CNNscore, and a predicted binding affinity (a p*K* value in negative log units) called CNNaffinity. There is also a third score, the CNN_VS score, which is the product of pose classification and predicted binding affinity. We use the CNN_VS score to rank compounds from docking as this was found to be effective in a retrospective virtual screening evaluation.^21^ We selected the top 1000 ligands with a molecular weight less than 550 Da, as ranked by their CNN_VS scores, for the next step of ensemble docking.

#### Pharmacophore Screening

We also obtained candidate ligands by performing a pharmacophore screening. A three-dimensional (3D) pharmacophore consists of the essential features of a molecular interaction and their spatial relationship.^22,23^ Common features considered include hydrophobic, aromatic, hydrogen-bonding, and charged interactions. This simplified representation of what a small-molecule binder should look like supports rapid and efficient search algorithms. We used the Pharmit^24^ online virtual screening resource, which uses the sublinear time Pharmer^25^ search algorithm, to screen millions of commercially available compounds in a few seconds.

Elucidating the correct pharmacophore for an interaction, especially when only the apo structure is available, remains an open problem, but docking-based solutions can successfully identify pharmacophores that produce subsets of compounds that are enriched for actives.^26,27^ Our pharmacophores were constructed by docking molecular fragments (benzene, acetate, acetamide, isobutane, isopropanol, isopropylamine, imidazole, guanidine, and water) to the central cavity of the crystal structure (PDB: 6DLO) and manually selecting the pharmacophore features of high scoring fragments with good interactions. We used Pharmit^24^ to screen the ZINC20, MCULE, and MCULE Ultimate databases (containing more than 179 million compounds) against 7 unique pharmacophores, each pharmacophore having 4 to 6 interaction features.

Pharmacophore search alone is not sufficient to rank compounds as it only identifies matches; an additional scoring step is required to rank compounds. Compounds were energy-minimized in Pharmit, and we filtered out compounds from the pharmacophore screen that had Vina scores greater (less negative) than −6.5 and compounds whose structural rearrangement upon Vina minimization was greater than 2 Å RMSD. We additionally applied a molecular weight filter to exclude all compounds with molecular weight exceeding 550 Da. This yielded 3572 compounds whose poses were further minimized and scored with the Gnina CNN scoring function.

##### Ensemble Docking

The top 1000 compounds from large-scale docking and 3572 compounds from pharmacophore screening were docked into the MD ensemble structures by using Gnina. For each compound and for each scoring function (Vina/Gnina), we retain the best observed score across the ensemble structures, including the crystal structure (PDB: 6DLO) that was the initial target of the screen.

##### Selection for Testing

The next step was to reduce our 4572 compounds obtained from docking and pharmacophore screening to a list of approximately 100 to be submitted for experimental testing. We sought to submit compounds from both pharmacophore screening and large-scale docking as well as the best compounds as determined by the AutoDock Vina and Gnina scoring functions. A ranked list was composed of interleaving the top compounds from Vina/Gnina and pharmacophore screening/docking. At this stage in the process, ENAMINE had reopened and the CACHE organizers, instead of merely suggesting ENAMINE as a supplier, now required all compounds to be sourced from ENAMINE. As there was not sufficient time to redo the screen against a new library, we adapted our results to the ENAMINE library by searching for ENAMINE compounds with a Tanimoto score of 1.0 to our identified compounds. We then applied diversity filtering to the ranked list of ENAMINE-available compounds; compounds were clustered using OpenBabel^17^ FP2 fingerprints and the list was pruned so that no more than two compounds were selected from each cluster.

Due to issues with ENAMINE availability, not all of the highest-scoring compounds from our initial screen were selected for testing. Only 25% of the 4572 compounds obtained from docking and pharmacophore screening were in the ENAMINE database. Additionally of the 109 compounds that were ordered from ENAMINE, 83 were actually successfully synthesized and tested. Our final selection ended up imbalanced: 59 ligands were obtained via large-scale docking, and 24 were obtained from pharmacophore screening.

### Round 2: Hit Expansion

Participants with identified hits from round 1 were invited to submit up to 50 follow-up compounds. For this round, we identified the 5000 most similar ligands to the hit compound with the best affinity in the ENAMINE REAL database as determined by Tanimoto similarity on Morgan Fingerprints computed using RDKit.^28^ The ENAMINE REAL database was filtered to exclude compounds with PAINs alerts and *c*log *P* > 4. The ligands found by a similarity search were then docked into the crystal structure using Gnina. We tried to select an equal mixture of compounds with the best Gnina and Vina scores for testing; the final selection ended up being imbalanced due to some compounds being unavailable. Of the 32 compounds experimentally evaluated in round 2, 22 were top-ranked by Gnina and 10 were top-ranked by Vina. As with round 1, no manual selection of hits was performed.

### Computational Resources for Screening

Screening was conducted on the University of Pittsburgh’s Computational and Systems Biology computational cluster. Computationally intensive tasks were parallelized across 4936 CPU cores as preemptable, low-priority jobs. The most time-intensive step in our screening pipeline was docking 7 million compounds from Molport for Round 1. The amount of time required for this task depends heavily on the level of concurrent cluster usage. Docking should take 2 weeks with light concurrent usage and 4–6 weeks with heavier concurrent usage. All screening was done within the 2-month window required by the CACHE Challenge organizers.

### MD Simulation of Hit Compounds

Molecular dynamics (MD) simulations were run on the three hit compounds obtained: CACHE_1181_33 and CACHE_1181_50 from Round 1 of the challenge and CACHE-HO_1181_24 from Round 2. Simulations were initialized with the docked ligand poses that led to their selection for experimental testing. Preparation of the protein as well as system equilibration were performed as with the structures used for ensemble docking. Ligands were parametrized using antechamber and the GAFF force field.^29^ For each ligand, three replicate 100 ns production simulations were performed.

## Results

Putative hits were evaluated experimentally by the CACHE organizers using surface plasmon resonance and the most promising hits were validated by at least one orthogonal direct binding assay, either isothermal titration calorimetry (ITC) or ^19^F NMR (full details are available elsewhere).^30^

### Round 1

**Figure 3:**
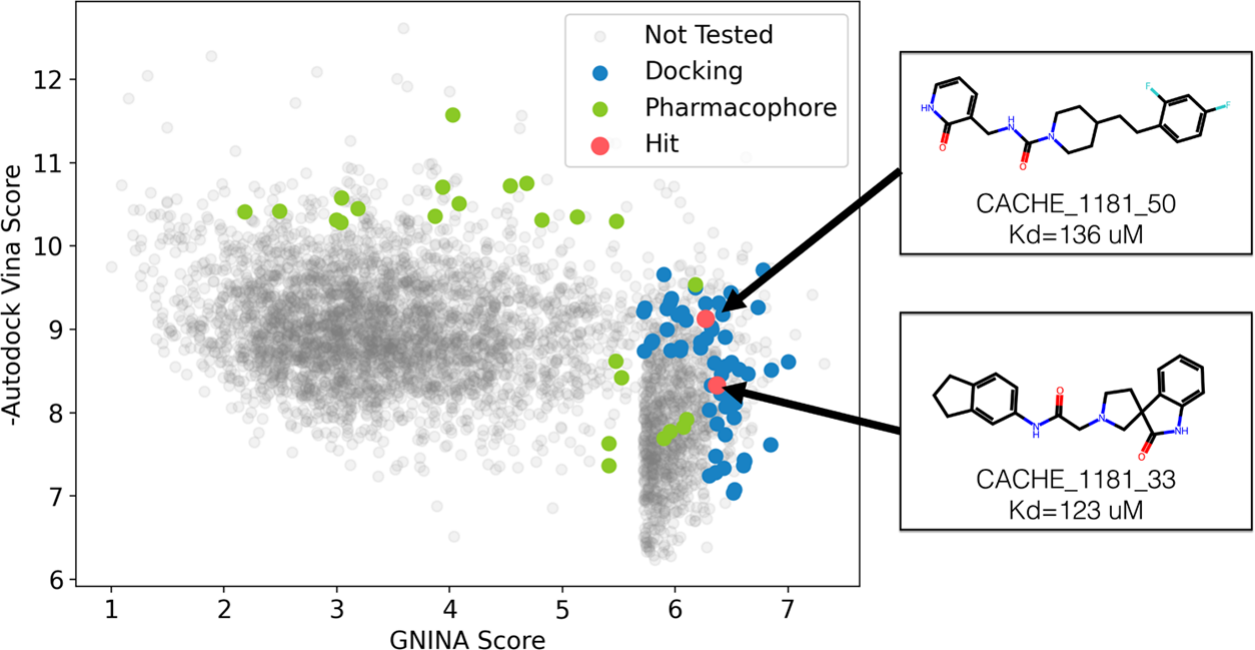
All compounds evaluated with ensemble docking were plotted using their AutoDock Vina and Gnina (CNN_VS) scores with experimentally tested compounds highlighted. Two of the compounds were considered hits by the CACHE organizers and are shown with their structures.

Of the 83 compounds from round 1 that were experimentally evaluated, two were identified by the CACHE organizers as potential hit compounds, although they had weak binding affinities with measured *K*_d_ values greater than 100 μM. These compounds are shown in Figure 3 plotted with respect to all of the top compounds that were evaluated using ensemble docking. The plotted scores are the maximum scores from ensemble docking and prior to filtering for diversity and ENAMINE availability, hence the acquired and tested compounds do not necessarily correspond to the top scores. Both hits from round 1 had comparatively higher Gnina scores than Vina scores and were identified by using molecular docking; no hits were identified from the pharmacophore screen.

#### Pharmacophore Screening vs Large-Scale Docking

Although both hits from round 1 were identified via large-scale docking, it is not possible to conclude that pharmacophore screening is less effective than large-scale docking due to the sample sizes. Our large-scale docking had a hit rate of ≈3.4% . If we assume that pharmacophore screening had the same hit rate, the expected number of hits is 0.72 and the probability of obtaining 0 hits from 24 tested compounds is ≈0.45. Our results presented here do not have enough statistical power to conclude that pharmacophore screening is more or less effective than large-scale docking.

**Figure 4:**
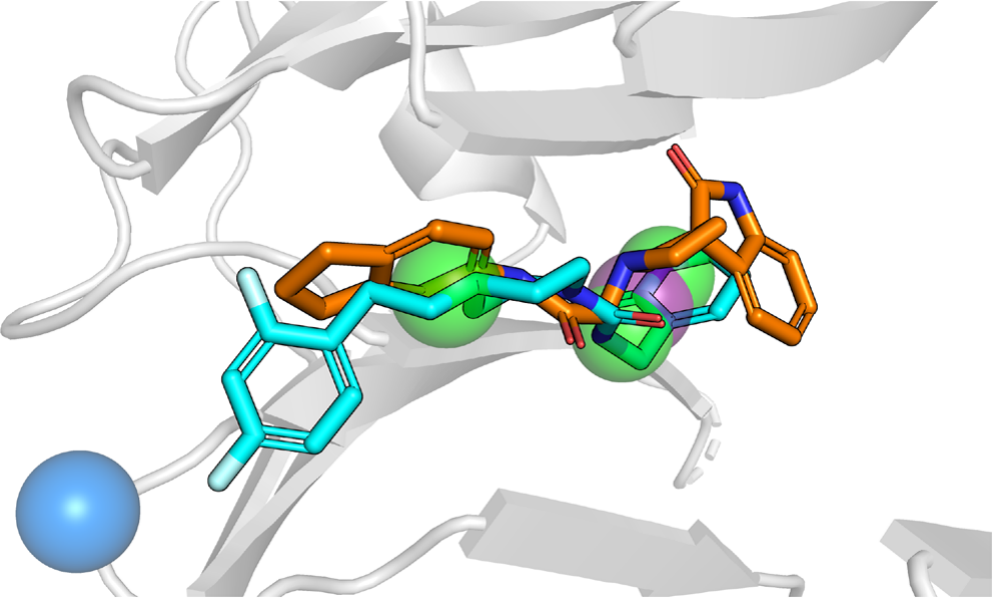
Transparent spheres indicate the pharmacophore features of the pharmacophore with the most experimentally tested matches (one of the seven pharmacophores that were used for screening). The blue sphere is a hydrogen bond donor group. Green spheres are hydrophobic groups. The purple sphere is an aromatic group. The orange and cyan ligands are hit compounds CACHE_1181_33 and CACHE_1181_50 in the docked poses that resulted in them being selected for experimental validation. The cartoon representation of 6DLO is shown in light gray.

We compare the pharmacophores used to obtain the compounds tested from pharmacophore screening with the docked poses that produced hit compounds in Figure 4. We observe some agreement between the pharmacophore features and the docked poses that produced the hit compounds. This result suggests that the pharmacophores used for virtual screening are not obviously implausible/ineffective and that they successfully recapitulate some of the molecular interactions identified by large-scale docking.

### Round 2

**Figure 5:**
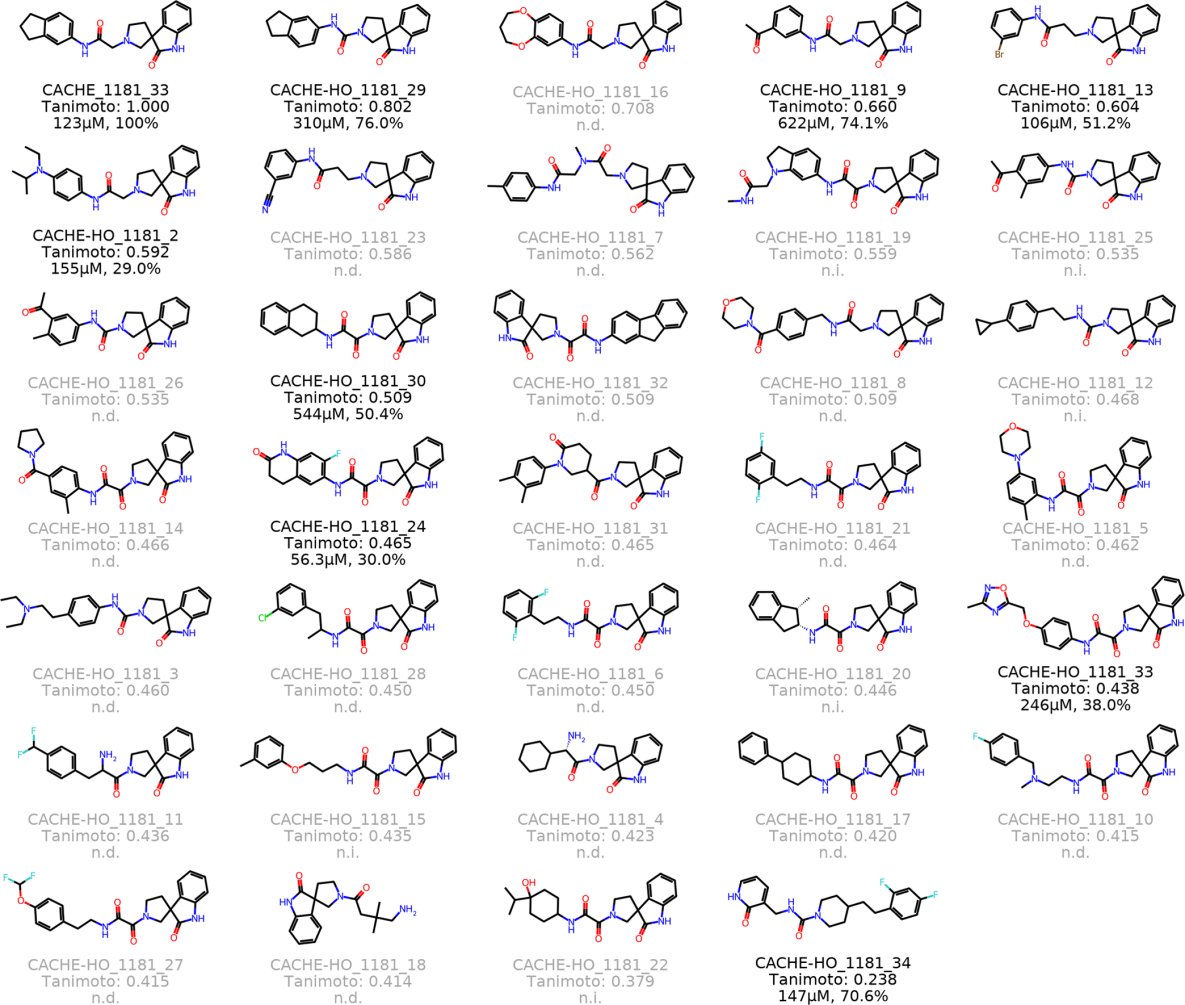
All compounds tested in the hit optimization (HO) round, sorted by their similarity to the parent compound, CACHE_1181_33. Tanimoto similarity is calculated between ECFP4 fingerprints. Compounds that did not exhibit binding in a primary single dose screen are labeled n.d. Compounds that were tested for a full dose response but did not exhibit statistically significant binding are labeled n.i. Otherwise, both the measured *K*_d_ and binding percentage are reported. Note that the other initial hit was also included as a hit optimization compound (CACHE-HO_1181_34) for retesting.

**Figure 6:**
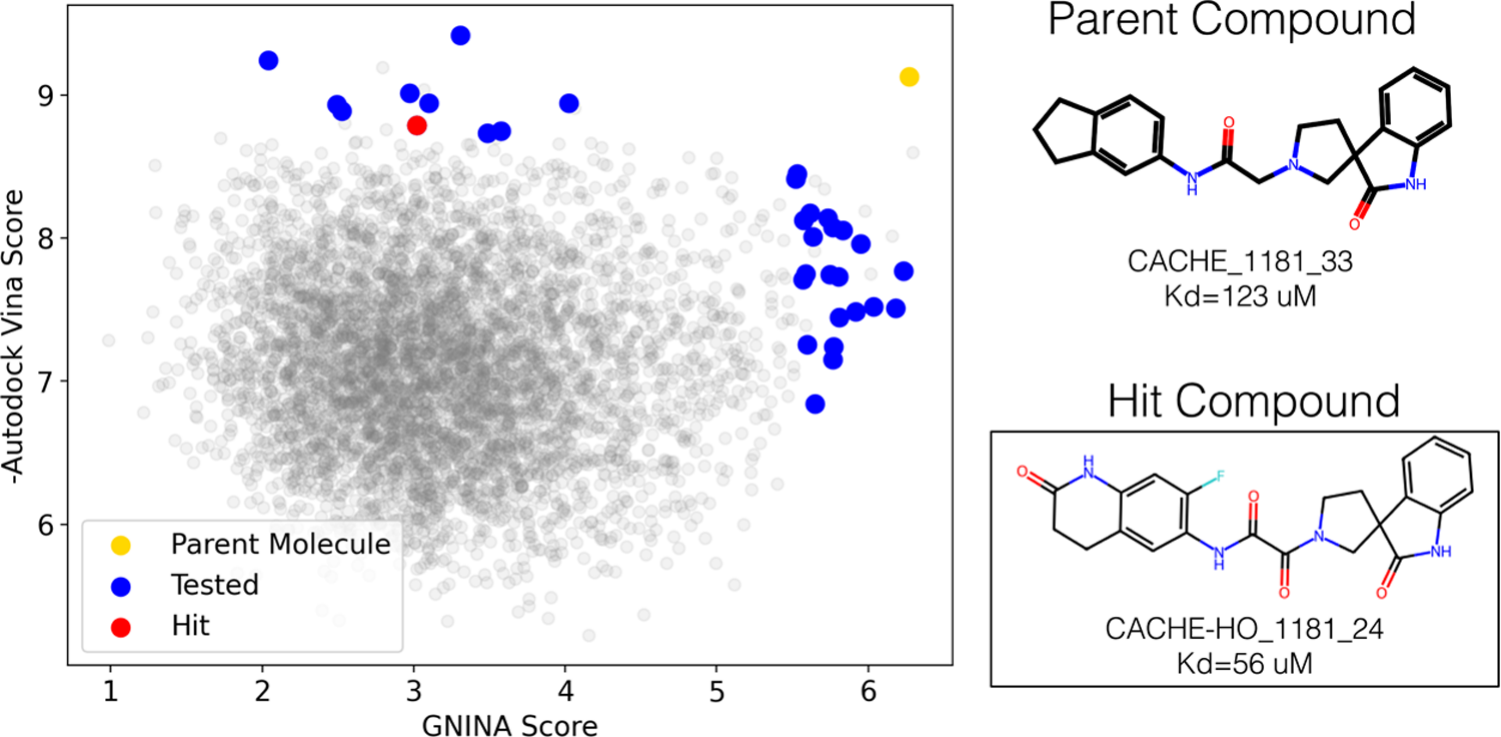
Distribution of docking scores for round 2 analogues of CACHE_1181_33.

For the hit expansion phase, we focused on the hit with slightly better *K*_d_, CACHE_1181_33. The docking scores of all identified analogues are shown in Figure 6. Of the compounds that were ultimately purchasable, the 10 compounds with the best Vina score and 22 compounds with the best Gnina (CNN_VS) score were acquired and evaluated. None of the analogues had better Gnina scores, which is perhaps unsurprising as the initial hit was selected based on having a top Gnina score (but from a subset of the full ENAMINE database from which the analogues were selected). Interestingly, the analogue that bound LRRK2 with the most improved affinity to the parent compound was identified based on its Vina score and had a *K*_d_ of 56 μM, as measured by surface plasmon resonance (SPR). As it is fluorinated, its binding was also confirmed by ^19^F NMR. All compounds submitted for the hit optimization phase are shown in Figure 5. It is noteworthy that compounds with a high Tanimoto similarity to the parent compound were more likely to demonstrate activity.

### Binding Mode and Protein–Ligand Interactions of Hit Compounds

**Figure 7:**
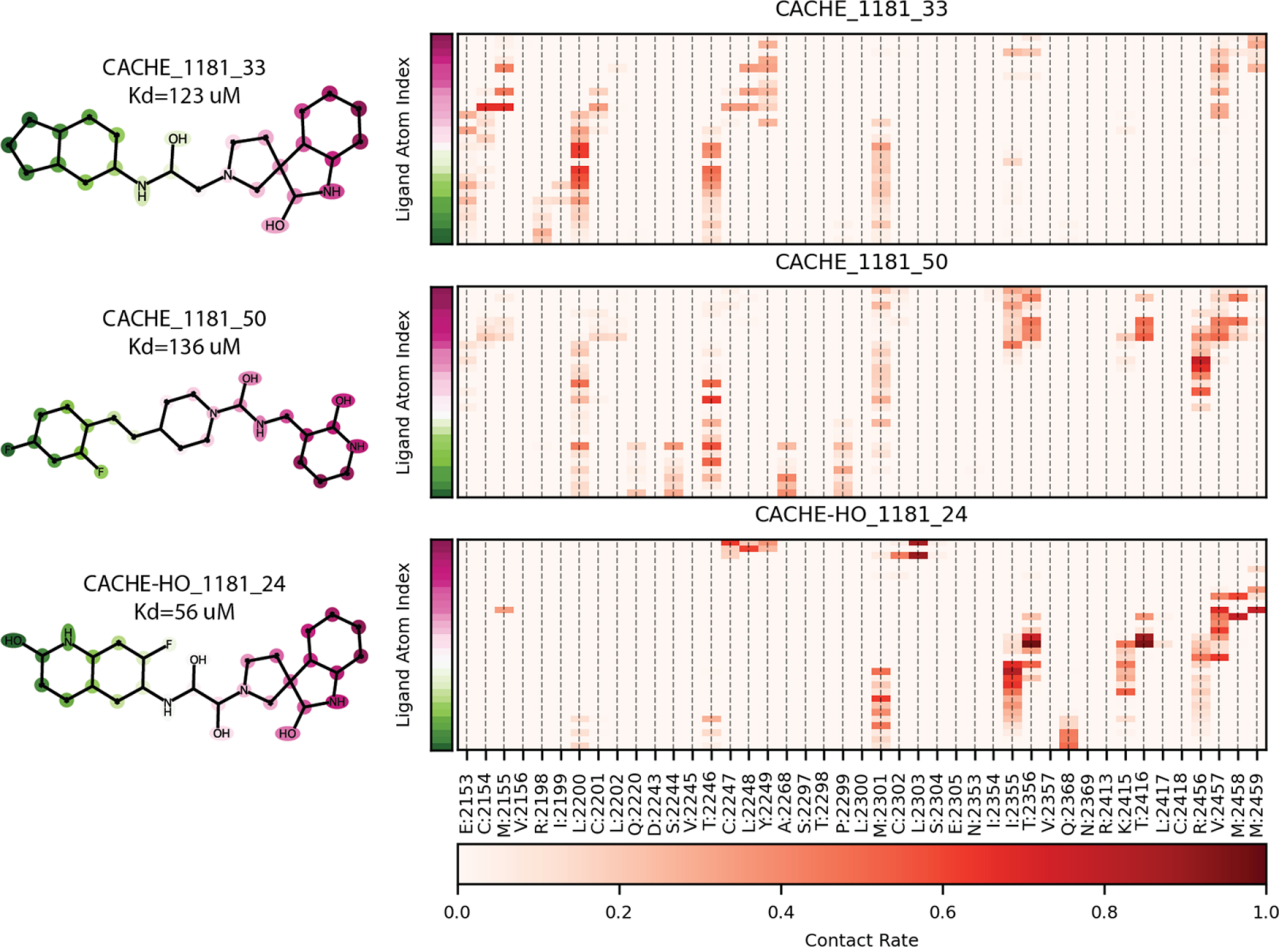
Protein ligand contact maps from the MD simulations. Contact maps are colored by contact rate, which is defined as the fraction of frames where a given residue has heavy atoms within 4 Å of a ligand heavy atom. The *x*-axis corresponds to protein residues and the *y*-axis corresponds to the heavy atoms of the ligand. Ligand atoms in the contact map are ordered according to their placement in the pocket. Magenta, or the top of the *y*-axis, is the end of the ligand buried in the pocket. Green, or the bottom of the *y*-axis, indicates the opposite end of the ligand that protrudes from the binding pocket. Ligand atoms in the 2D structures on the left of the figure are colored by this ordering as well.

To better understand the putative molecular interactions and the role of receptor flexibility, we performed molecular dynamics simulations of the hit compounds bound to the WDR domain. Figure 7 shows the prevalence of contacts between heavy ligand atoms and protein residues in these simulations. The contact maps display the contact frequency: the fraction of MD frames where ligand and protein heavy atoms have an interatomic distance less than 4 Å. The contact maps of Figure 7 suggest that CACHE-HO_1181_24 forms more stable and consistently observed contacts with the protein. This pattern is consistent with the wet lab measurements of binding affinity.

**Figure 8:**
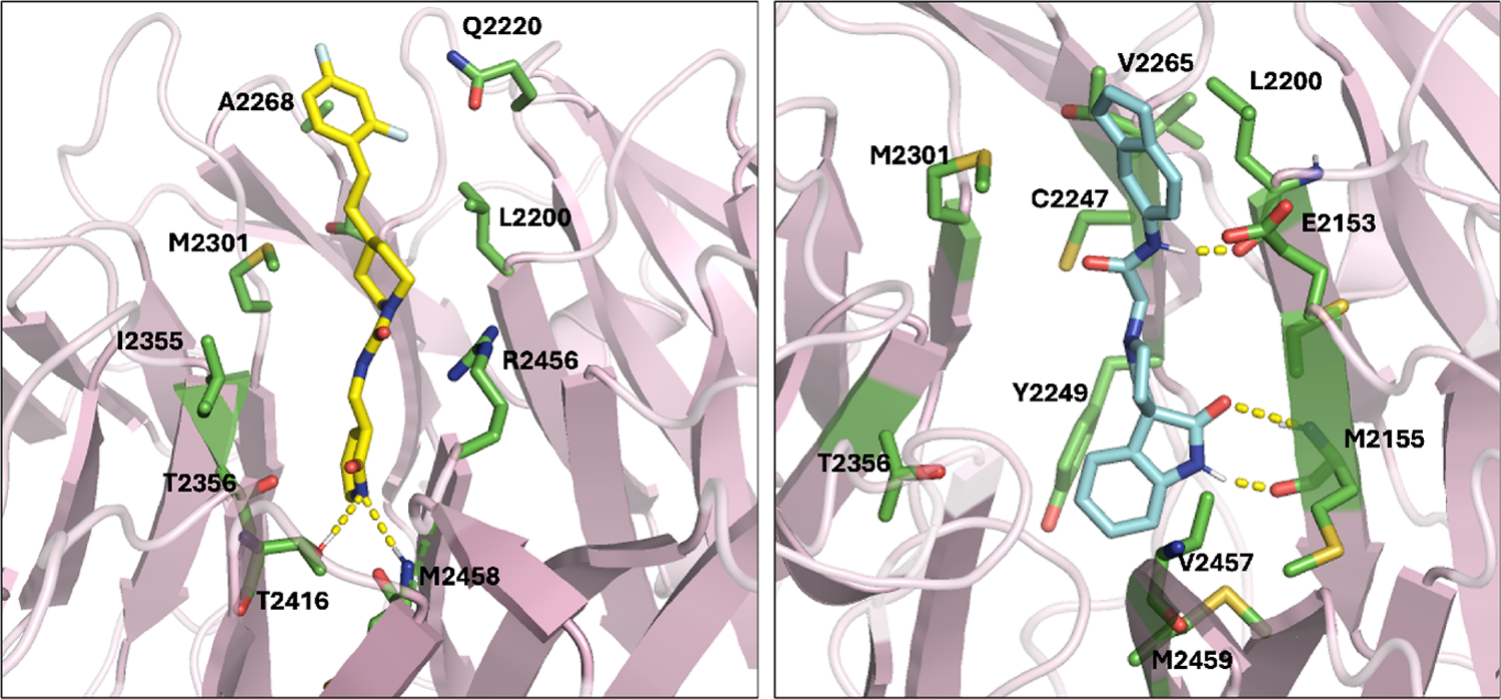
Representative bound pose for CACHE_1181_33 (left) and CACHE_1181_50 (right). All residues that have heavy atoms within 4 Å of the ligand are labeled. Hydrogen bonds are depicted with dashed yellow lines.

**Figure 9:**
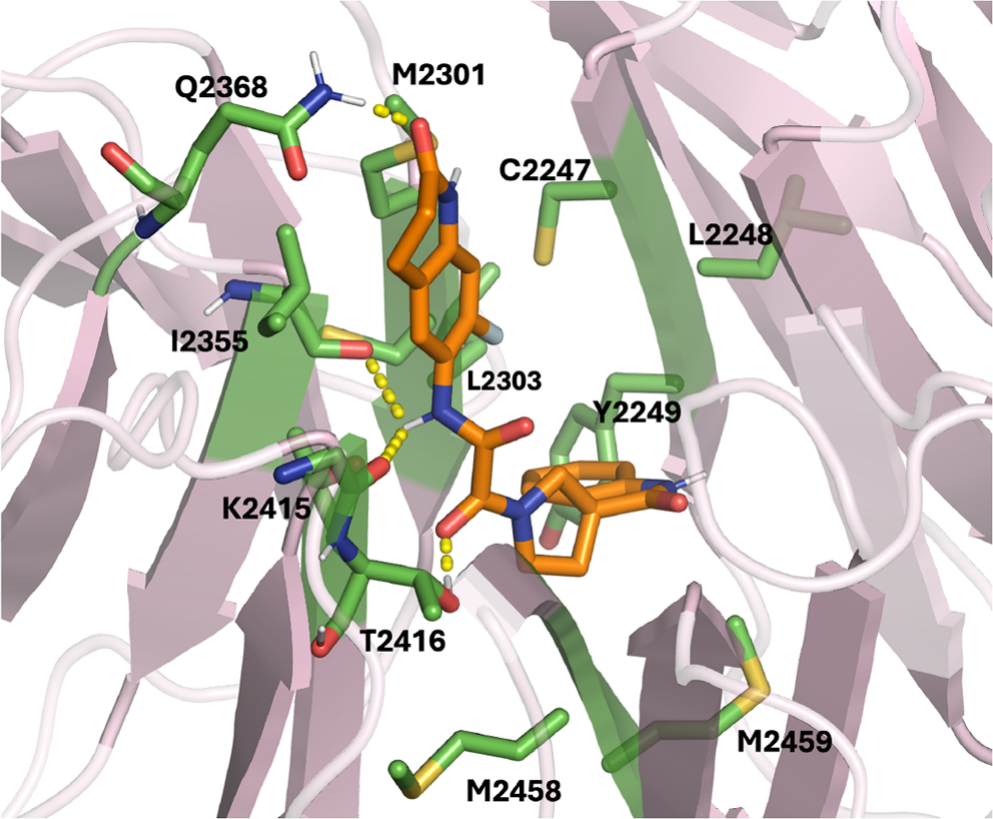
Representative bound pose for CACHE-HO_1181_24. All residues that have heavy atoms within 4 Å of the ligand are labeled. Hydrogen bonds are depicted with dashed yellow lines.

The hit compounds are linear and bind vertically into the central cavity of the WDR domain, with one end buried in the cavity and the other protruding toward the outside of the cavity, as shown in the representative poses in Figures 8 and 9. The protruding ends of CACHE_1181_33 and CACHE_1181_50 appear to make intermittent contact with several residues. The buried rings in CACHE_1181_33 and CACHE_1181_50 contain heteroatoms that form hydrogen bonds with protein residues that keep these regions relatively stable while the protruding ends of the ligands move more freely. The buried end of CACHE_1181_33 forms a hydrogen bond with M2155. CACHE_1181_50 forms hydrogen bonds with T2416 and M2458. Representative binding poses illustrating these interactions are shown in Figure 8.

The middle region of CACHE-HO_1181_24 forms hydrogen bonds with I2355 and K2415. Notably, the protruding end of the ligand forms a hydrogen bond, Q2368, resulting in this end of the ligand being substantially more stabilized than the same regions of CACHE_1181_33 and CACHE_1181_50. A representative binding pose is shown in Figure 9.

### Receptor Flexibility Analysis

During Round 1 screening, an ensemble of apo protein structures was used for docking. Here, we use bound poses from MD simulations with the hit ligands to assess the presence and effect of receptor flexibility in the ensemble docking procedure. From MD trajectories, we define a set of “binding site residues” as the set of residues that form contact with a ligand atom for at least 30% of the simulation frames. Our analysis of protein conformational changes is constrained to these binding site residues.

**Figure 10:**
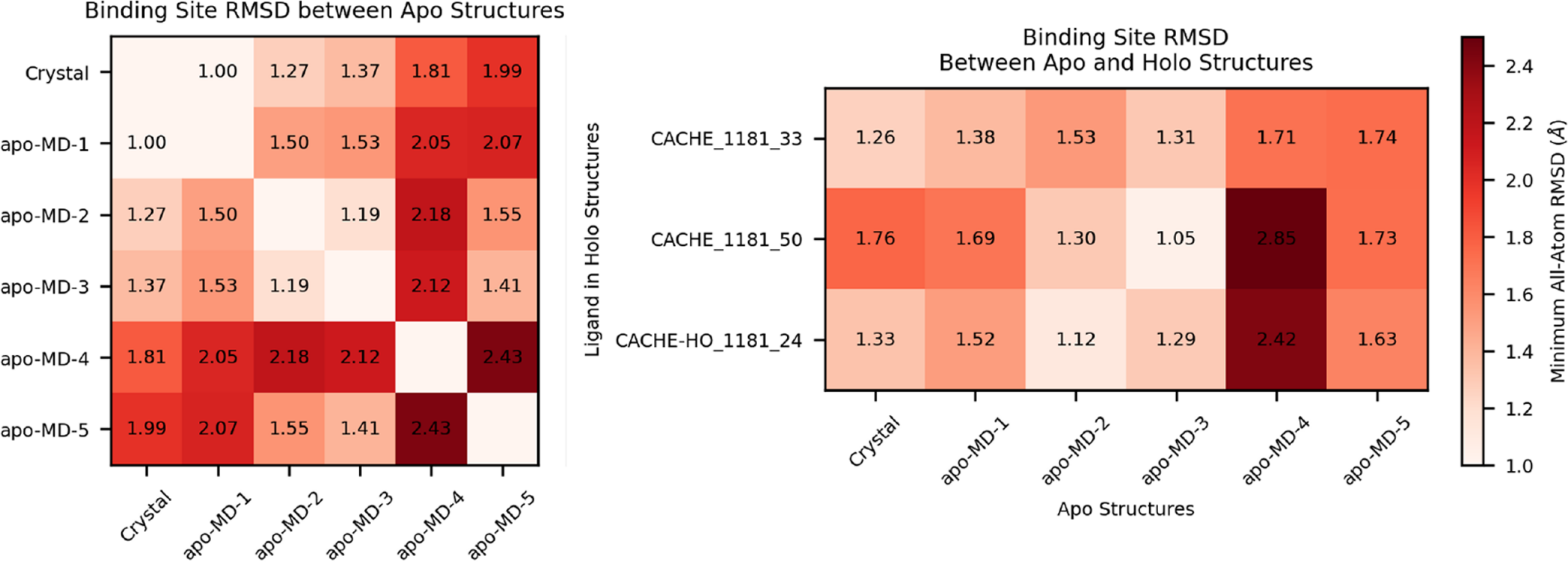
Left: Pairwise, minimum, all-atom RMSD of binding residues across apo structures used for screening. Right: RMSD calculation used for apo structures applied to pairs of holo structures from MD simulations and the apo structures used for screening.

We computed the pairwise all-atom RMSD of the binding site residues in apo structures used for docking in Round 1. These apo structures are the crystal structure 6DLO and an ensemble of 5 representative frames from MD simulation of the apo structure. The pairwise RMSDs are displayed in Figure 10. Note that we computed the RMSD after rigid-body alignment for each pair. The RMSD values between apo structures suggest that there are relatively small rearrangements of the protein–ligand interface across the apo structures. In the most distant pair of apo structures, an RMSD of 2.4 Å corresponded to the side chains of just a few solvent-exposed residues changing direction.

We approximate holo structures using representative frames from ligand-bound MD simulations. We chose 5 representative MD frames for each ligand; chosen as the frames with the most high-frequency contacts. A high-frequency contact was defined as a contact between a residue and specific ligand atom that is observed in more than 30% of frames. We compute the all-atom minimum RMSD between holo structures from MD simulations and the apo structures used for screening. The results in Figure 10 demonstrate that the MD-generated apo structures contained binding site conformations closer to the holo structure than the original apo crystal structure for 2/3 hit ligands. However, the similarity to holo structures does not correlate well with the docking scores obtained for that apo structure. For example, CACHE_1181_33 and CACHE_1181_50 had the best docking scores in the crystal and apo-MD-2 structures, respectively. According to the Apo-Holo RMSD values in Figure 10, the apo structures with the best docking scores were not the most similar to the holo binding site structure. This could be because the conformational heterogeneity of the apo ensemble is relatively limited, the apo structures are all relatively close to the holo conformation, or because the GNINA CNN scoring function is robust to small side chain rearrangements by design.

## Discussion

The field of computational drug discovery is undergoing a deep-learning-powered revolution. The convolutional neural networks underlying Gnina were some of the first deep neural networks designed for structure-based drug discovery and yet are relatively simplistic compared to more recent end-to-end approaches to deep molecular docking,^31−33^ including approaches that incorporate full protein flexibility.^34,35^ However, a common pitfall of all learning-based approaches is that it is difficult to determine how well models generalize to novel instances; apparent high performance in retrospective studies can be an artifact of biases in data set construction,^36−38^ and the resulting models can severely underperform more conventional approaches in real-world applications. This underscores the importance of the CACHE exercise, as it provides a blinded and prospective evaluation of virtual screening methods.

The first CACHE competition featured participation by 23 groups. Of these, 18 advanced to the hit expansion round. At the conclusion of the exercise, the resulting data was reviewed by an independent Hit Evaluation Committee, and the most promising lead series were assigned an aggregate score. Our submission tied with another^39^ for the highest score. Although limited, the reviewers found the structure–activity relationship around our identified compound (CACHE_1181_33) to be compelling with the potential to be further developed through accessible analogues. It is worth noting that although our submission was rated highly, we did not have the highest hit rate (only 2.3% in round 1, compared to a competition average of 3.7%) nor the best measured affinity (the best compound had a *K*_d_ of 54 μM). Interestingly, our compounds were rated highly in this subjective judging exercise even though no manual curation was performed to select the compounds—all selections were purely based on docking scores, cheminformatic properties, and commercial availability. This is in contrast to many of the other submissions which included manual curation and/or more advanced ranking approaches such as FEP.^39^

The WDR domain was a particularly difficult target; there are no known ligands, and it is unlikely that the central cavity has evolved to bind to small molecules. Thus, it is not surprising that overall, there were low hit rates, even for a generous definition of a hit, and the hits identified were weak binders. The small number of identified hits makes it difficult to draw statistically meaningful conclusions when comparing the different approaches. However, we find it noteworthy that a purely docking-based approach (with Gnina) was not obviously distinguishable from more complex approaches that used molecular simulation or were guided by expert medicinal chemists. We look forward to the outcome of future CACHE challenges and anticipate that as more data are collected across a variety of targets and more methods are evaluated, a clearer picture will emerge of the state of the art in computational drug discovery and to what extent emerging deep learning methods can accelerate the process.

## Data Availability

Our entire workflow is open source (AMBER is partially open source; GROMACS is a fully open-source alternative). Gnina is available at https://github.com/gnina. Chemical structures and experimental results for all CACHE submissions are available at https://cache-challenge.org/results-cache-challenge-1.

## References

[ref1] Li F.; Ackloo S.; Arrowsmith C. H.; et al. CACHE Challenge #1: targeting the WDR domain of LRRK2, a Parkinson’s Disease associated protein. J. Chem. Inf. Model. 2024, 64 (22), 8521–8536. 10.1021/acs.jcim.4c01267. 39499532

[ref2] Berwick D. C.; Heaton G. R.; Azeggagh S.; Harvey K. LRRK2 Biology from structure to dysfunction: research progresses, but the themes remain the same. Mol. Neurodegener. 2019, 14, 49 10.1186/s13024-019-0344-2. 31864390 PMC6925518

[ref3] West A. B.; Moore D. J.; Biskup S.; Bugayenko A.; Smith W. W.; Ross C. A.; Dawson V. L.; Dawson T. M. Parkinson’s disease-associated mutations in leucine-rich repeat kinase 2 augment kinase activity. Proc. Natl. Acad. Sci. U.S.A. 2005, 102, 16842–16847. 10.1073/pnas.0507360102. 16269541 PMC1283829

[ref4] Hatcher J. M.; Zwirek M.; Sarhan A. R.; Vatsan P. S.; Tonelli F.; Alessi D. R.; Davies P.; Gray N. S. Development of a highly potent and selective degrader of LRRK2. Bioorg. Med. Chem. Lett. 2023, 94, 129449 10.1016/j.bmcl.2023.129449. 37591317 PMC11837620

[ref5] Liu X.; Kalogeropulou A. F.; Domingos S.; Makukhin N.; Nirujogi R. S.; Singh F.; Shpiro N.; Saalfrank A.; Sammler E.; Ganley I. G.; Moreira R.; Alessi D. R.; Ciulli A. Discovery of XL01126: A Potent, Fast, Cooperative, Selective, Orally Bioavailable, and Blood-Brain Barrier Penetrant PROTAC Degrader of Leucine-Rich Repeat Kinase 2. J. Am. Chem. Soc. 2022, 144, 16930–16952. 10.1021/jacs.2c05499. 36007011 PMC9501899

[ref6] Schapira M.; Tyers M.; Torrent M.; Arrowsmith C. H. WD40 repeat domain proteins: a novel target class?. Nat. Rev. Drug Discovery 2017, 16, 773–786. 10.1038/nrd.2017.179. 29026209 PMC5975957

[ref7] Zhang P.; Fan Y.; Ru H.; Wang L.; Magupalli V. G.; Taylor S. S.; Alessi D. R.; Wu H. Crystal structure of the WD40 domain dimer of LRRK2. Proc. Natl. Acad. Sci. U.S.A. 2019, 116, 1579–1584. 10.1073/pnas.1817889116. 30635421 PMC6358694

[ref8] Myasnikov A.; Zhu H.; Hixson P.; Xie B.; Yu K.; Pitre A.; Peng J.; Sun J. Structural analysis of the full-length human LRRK2. Cell 2021, 184, 3519–3527. 10.1016/j.cell.2021.05.004. 34107286 PMC8887629

[ref9] McNutt A. T.; Francoeur P.; Aggarwal R.; Masuda T.; Meli R.; Ragoza M.; Sunseri J.; Koes D. R. GNINA 1.0: molecular docking with deep learning. J. Cheminf. 2021, 13, 43 10.1186/s13321-021-00522-2. PMC8191141 34108002

[ref10] Ragoza M.; Hochuli J.; Idrobo E.; Sunseri J.; Koes D. R. Protein–ligand scoring with convolutional neural networks. J. Chem. Inf. Model. 2017, 57, 942–957. 10.1021/acs.jcim.6b00740. 28368587 PMC5479431

[ref11] Case D. A.; Cheatham T. E. III; Darden T.; Gohlke H.; Luo R.; Merz K. M. Jr.; Onufriev A.; Simmerling C.; Wang B.; Woods R. J. The Amber biomolecular simulation programs. J. Comput. Chem. 2005, 26, 1668–1688. 10.1002/jcc.20290. 16200636 PMC1989667

[ref12] Case D. A.; Aktulga H. M.; Belfon K.; Cerutti D. S.; Cisneros G. A.; Cruzeiro V. W. D.; Forouzesh N.; Giese T. J.; Götz A. W.; Gohlke H.; et al. AmberTools. J. Chem. Inf. Model. 2023, 63, 6183–6191. 10.1021/acs.jcim.3c01153. 37805934 PMC10598796

[ref13] Debiec K. T.; Cerutti D. S.; Baker L. R.; Gronenborn A. M.; Case D. A.; Chong L. T. Further along the Road Less Traveled: AMBER ff15ipq, an Original Protein Force Field Built on a Self-Consistent Physical Model. J. Chem. Theory Comput. 2016, 12, 3926–3947. 10.1021/acs.jctc.6b00567. 27399642 PMC4980686

[ref14] Koes D. R.; Vries J. K. Evaluating amber force fields using computed NMR chemical shifts. Proteins: Struct., Funct., Bioinf. 2017, 85, 1944–1956. 10.1002/prot.25350. PMC6193454 28688107

[ref15] Koes D. md-scripts/diversemdselect.py at master·dkoes/md-scripts. https://github.com/dkoes/md-scripts/blob/master/diversemdselect.py.

[ref16] Ragoza M.; Masuda T.; Koes D. R. Learning a Continuous Representation of 3D Molecular Structures with Deep Generative Models, arXiv:2010.08687. arXiv.org e-Print archive, 2020. https://arxiv.org/abs/2010.08687.

[ref17] O’Boyle N. M.; Banck M.; James C. A.; Morley C.; Vandermeersch T.; Hutchison G. R. Open Babel: An open chemical toolbox. J. Cheminf. 2011, 3, 33 10.1186/1758-2946-3-33. PMC3198950 21982300

[ref18] Koes D. R.; Baumgartner M. P.; Camacho C. J. Lessons learned in empirical scoring with smina from the CSAR 2011 benchmarking exercise. J. Chem. Inf. Model. 2013, 53, 1893–1904. 10.1021/ci300604z. 23379370 PMC3726561

[ref19] Eberhardt J.; Santos-Martins D.; Tillack A. F.; Forli S. AutoDock Vina 1.2.0: New Docking Methods, Expanded Force Field, and Python Bindings. J. Chem. Inf. Model. 2021, 61, 3891–3898. 10.1021/acs.jcim.1c00203. 34278794 PMC10683950

[ref20] Sunseri J.; Koes D. R. Libmolgrid: graphics processing unit accelerated molecular gridding for deep learning applications. J. Chem. Inf. Model. 2020, 60, 1079–1084. 10.1021/acs.jcim.9b01145. 32049525 PMC7500858

[ref21] Sunseri J.; Koes D. R. Virtual screening with Gnina 1.0. Molecules 2021, 26, 7369 10.3390/molecules26237369. 34885952 PMC8659095

[ref22] Koes D. R. Pharmacophore Modeling: Methods and Applications. In Methods in Pharmacology and Toxicology; Springer, 2016; pp 167–188.

[ref23] Leach A. R.; Gillet V. J.; Lewis R. A.; Taylor R. Three-dimensional pharmacophore methods in drug discovery. J. Med. Chem. 2010, 53, 539–558. 10.1021/jm900817u. 19831387

[ref24] Sunseri J.; Koes D. R. Pharmit: interactive exploration of chemical space. Nucleic Acids Res. 2016, 44, W442–W448. 10.1093/nar/gkw287. 27095195 PMC4987880

[ref25] Koes D. R.; Camacho C. J. Pharmer: efficient and exact pharmacophore search. J. Chem. Inf. Model. 2011, 51, 1307–1314. 10.1021/ci200097m. 21604800 PMC3124593

[ref26] Mortier J.; Dhakal P.; Volkamer A. Truly target-focused pharmacophore modeling: a novel tool for mapping intermolecular surfaces. Molecules 2018, 23, 1959 10.3390/molecules23081959. 30082611 PMC6222449

[ref27] Heider J.; Kilian J.; Garifulina A.; Hering S.; Langer T.; Seidel T. Apo2ph4: a versatile workflow for the generation of receptor-based pharmacophore models for virtual screening. J. Chem. Inf. Model. 2023, 63, 101–110. 10.1021/acs.jcim.2c00814. 36526584 PMC9832483

[ref28] RDKit. http://www.rdkit.org/.

[ref29] Wang J.; Wolf R. M.; Caldwell J. W.; Kollman P. A.; Case D. A. Development and testing of a general amber force field. J. Comput. Chem. 2004, 25, 1157–1174. 10.1002/jcc.20035. 15116359

[ref30] Results of CACHE Challenge #1, 2024. https://cache-challenge.org/results-cache-challenge-1. Accessed: July 16, 2024.

[ref31] Corso G.; Stärk H.; Jing B.; Barzilay R.; Jaakkola T. Diffdock: Diffusion steps, twists, and turns for molecular docking, arXiv:2210.01776. arXiv.org e-Print archive, 2022. https://arxiv.org/abs/2210.01776.

[ref32] Stark H.; Jing B.; Barzilay R.; Jaakkola T. In Harmonic Self-Conditioned Flow Matching for joint Multi-Ligand Docking and Binding Site Design, Forty-First International Conference on Machine Learning; ICML, 2024.

[ref33] Brocidiacono M.; Popov K. I.; Koes D. R.; Tropsha A. Plantain: diffusion-inspired pose score minimization for fast and accurate molecular docking ArXiv 2023.

[ref34] Lu W.; Zhang J.; Huang W.; Zhang Z.; Jia X.; Wang Z.; Shi L.; Li C.; Wolynes P. G.; Zheng S. DynamicBind: Predicting ligand-specific protein-ligand complex structure with a deep equivariant generative model. Nat. Commun. 2024, 15, 1071 10.1038/s41467-024-45461-2. 38316797 PMC10844226

[ref35] Abramson J.; Adler J.; Dunger J.; Evans R.; Green T.; Pritzel A.; Ronneberger O.; Willmore L.; Ballard A. J.; Bambrick J.; et al. Accurate structure prediction of biomolecular interactions with AlphaFold 3. Nature 2024, 630, 493–500. 38718835 10.1038/s41586-024-07487-w PMC11168924

[ref36] Chen L.; Cruz A.; Ramsey S.; Dickson C. J.; Duca J. S.; Hornak V.; Koes D. R.; Kurtzman T. Hidden bias in the DUD-E dataset leads to misleading performance of deep learning in structure-based virtual screening. PLoS One 2019, 14, e0220113 10.1371/journal.pone.0220113. 31430292 PMC6701836

[ref37] Sieg J.; Flachsenberg F.; Rarey M. In need of bias control: evaluating chemical data for machine learning in structure-based virtual screening. J. Chem. Inf. Model. 2019, 59, 947–961. 10.1021/acs.jcim.8b00712. 30835112

[ref38] Durairaj J.; Adeshina Y.; Cao Z.; Zhang X.; Oleinikovas V.; Duignan T.; McClure Z.; Robin X.; Kovtun D.; Rossi E. PLINDER: The protein-ligand interactions dataset and evaluation resource bioRxiv 2024.

[ref39] Gutkin E.; Gusev F.; Gentile F.; Ban F.; Koby S. B.; Narangoda C.; Isayev O.; Cherkasov A.; Kurnikova M. G. In silico screening of LRRK2 WDR domain inhibitors using deep docking and free energy simulations. Chem. Sci. 2024, 15, 8800–8812. 10.1039/D3SC06880C. 38873063 PMC11168082

